# Adverse pregnancy and infant outcomes by COVID‐19 infection status before and during pregnancy

**DOI:** 10.1002/pmf2.70051

**Published:** 2025-06-30

**Authors:** Kelly J. Hunt, Chun‐Che Wen, Kalyan J. Chundru, Julio Mateus, Hermes Florez, Jeffrey E. Korte, Brian Neelon, Dulaney A. Wilson, John Pearce, Mallory H. Alkis, Sarah Simpson, Erin Alsbrook, Veronica Spates, Angela M. Malek

**Affiliations:** ^1^ Department of Public Health Sciences Medical University of South Carolina Charleston South Carolina USA; ^2^ Ralph H. Johnson Department of Veterans Affairs Medical Center Health Equity and Rural Outreach Innovation Center Charleston South Carolina USA; ^3^ Department of Obstetrics & Gynecology Maternal‐Fetal Medicine Division Atrium Health Charlotte North Carolina USA; ^4^ Department of Obstetrics & Gynecology Maternal‐Fetal Medicine Division Medical University of South Carolina Charleston South Carolina USA

**Keywords:** COVID‐19, maternal and infant outcomes, pandemic, pregnancy, SARS‐CoV‐2, trimester

## Abstract

**Introduction:**

To examine the association between pre‐pregnancy and gestational COVID‐19 infection by trimester with adverse pregnancy and infant outcomes.

**Methods:**

A retrospective cohort study was conducted to evaluate singleton live births and fetal deaths among pregnant women in South Carolina (SC) from January 2020 through December 2022. Birth certificate and COVID‐19 diagnosis data obtained from the SC Department of Health and Environmental Control were linked to inpatient hospital discharge and emergency department records and Medicaid eligibility files from the SC Revenue and Fiscal Affairs Office. Log‐binomial and modified Poisson models were used in matched and unmatched analyses to estimate risk ratios (RRs) for maternal and infant outcomes associated with COVID‐19 infection pre‐pregnancy, during the first/second trimester, and during the third trimester.

**Results:**

Of 145,028 pregnancies, first infection with COVID‐19 occurred for 7.5% before pregnancy, 5.5% during the first/second trimester, and 3.4% during the third trimester. In matched analyses following adjustment for sociodemographic, clinical, and behavioral factors, COVID‐19 infection pre‐pregnancy increased the risk for hypertensive disorders of pregnancy (HDP) (RR = 1.07, 95% CI: 1.03–1.14), preeclampsia/eclampsia (RR = 1.09, 95% CI: 1.01–1.17), and cesarean delivery (RR = 1.06, 95% CI: 1.00–1.11). COVID‐19 infection during the first/second trimester increased the risk for preeclampsia/eclampsia (RR = 1.10, 95% CI: 1.00–1.20) as well as placental abruption (RR = 1.15, 95% CI: 1.01–1.30) and severe maternal morbidity (SMM) with (RR = 1.60, 95% CI: 1.32–1.93) and without transfusion (RR = 1.91, 95% CI: 1.52–2.40) after full adjustment. COVID‐19 during the third trimester increased the risk for preeclampsia/eclampsia (RR = 1.15, 95% CI: 1.02–1.29), placenta abruption (RR = 1.38, 95% CI: 1.15–1.66), postpartum hemorrhage (RR = 1.24, 95% CI: 1.02–1.51), and SMM with (RR = 2.07, 95% CI: 1.65–2.59) and without transfusion (RR = 2.56, 95% CI: 1.98–3.35) after full adjustment.

**Conclusion:**

This study demonstrated that COVID‐19 infection during pregnancy and more notably before pregnancy was significantly associated with multiple adverse pregnancy events, after controlling for pertinent sociodemographic, clinical, and behavioral factors.

**Keypoints:**

- While prior studies have examined potential risks associated with COVID‐19 infection during pregnancy, our study is one of the first to investigate potential risks associated with COVID‐19 prior to pregnancy. We report significantly increased risk of hypertensive disorders of pregnancy (HDP), specifically preeclampsia/eclampsia, among pregnant women who had COVID‐19 infection prior to pregnancy compared to those without COVID‐19 in a statewide matched analyses after adjustment for covariates.
- COVID‐19 infection during the first or second trimester was associated with increased risk for preeclampsia/eclampsia, placental abruption, and severe maternal morbidity (SMM) relative to those with no history of COVID‐19.
- COVID‐19 infection during the third trimester was associated with increased risk of preeclampsia/eclampsia, placental abruption, postpartum hemorrhage, and SMM (both including and excluding transfusion).

## 1 INTRODUCTION

Since 2020, there have been over 6.7 million hospitalizations (August 1, 2020 to August 3, 2024) and 1.2 million deaths (January 1, 2020 to June 30, 2024) due to coronavirus disease 2019 (COVID‐19) in the United States (U.S.) [1]. Pregnant women are at higher risk of contracting COVID‐19 as well as experiencing more severe clinical manifestations predisposing them to multiple complications [2, 3]. Some past studies including three international meta‐analyses reported associations between COVID‐19 during pregnancy and adverse maternal and infant outcomes although only one small retrospective cohort study to date has assessed timing of infection prior to and during pregnancy [4]. Simbar et al.’s 2023 meta‐analysis of 74 studies reported a higher prevalence of preterm delivery (PTD), maternal mortality, neonatal intensive care unit (NICU) admission, and neonatal death associated with COVID‐19 infection during pregnancy [2]. Jeong and Kim's 2023 meta‐analysis, which included 69 studies comprising over 1.6 million women (39,716 with COVID‐19 during pregnancy), reported associations between COVID‐19 during pregnancy with a higher risk of PTD, preeclampsia, low birth weight, cesarean delivery (C‐section), stillbirth, fetal distress, NICU admission, perinatal mortality, and maternal mortality [3]. Another meta‐analysis from 2021 of 42 studies found relationships between COVID‐19 during pregnancy and preeclampsia, stillbirth, and PTD, with severe infection (vs. mild) strongly associated with preeclampsia, gestational diabetes (GDM), PTD, and low birth weight without examination by timing of trimester [5].

We are aware of one prior study examining the impact of COVID‐19 infection before pregnancy on pregnancy‐related outcomes [4]. However, it is an important time window of exposure to consider as COVID‐19 infection before conception and in general could change the mother's immune, respiratory, and cardiovascular systems and in turn negatively affect the physiological adaptations of pregnancy including placental development [4]. In this small retrospective cohort study conducted in St. Louis involving 71 pregnancies with (*n* = 52) and without (*n* = 19) COVID‐19 during or before pregnancy, 11 were infected with mild COVID‐19 pre‐conception (median = 97 days; range = 12–399) [4]. Compared to women without COVID‐19, those with infection occurring pre‐conception had lower birth weights and gestational ages at delivery, and were more likely to experience pregnancy loss before 20 weeks [4]. Moreover, multiple studies have indicated that COVID‐19 infection has potential long‐term implications on the cardiovascular system which in turn could impact pregnancy outcomes as pregnancy is dependent upon the placental vasculature as well as the maternal cardiovascular system [6, 7, 8, 9]. Hence, mechanisms linking COVID‐19 infection to maternal and infant outcomes are likely dependent upon timing of COVID‐19 infection in relation to pregnancy onset and trimester. Therefore, we examined the association between maternal COVID‐19 infection based on trimester of exposure or COVID‐19 onset before pregnancy and pregnancy outcomes in women who delivered from 2020 through 2022.

## 2 MATERIALS AND METHODS

### 2.1 Study design and population

A retrospective cohort study was conducted of singleton live births and fetal deaths among pregnant women in South Carolina (SC) from January 1, 2020, through December 31, 2022. Birth certificate and COVID‐19 diagnosis data obtained from the SC Department of Health and Environmental Control (DHEC) were linked to statewide inpatient hospital discharge and emergency department (ED) records and Medicaid eligibility files by the SC Revenue and Fiscal Affairs (RFA) Office. These included inpatient and ED procedure and diagnostic codes for the mother and the baby. As part of the integrated data system created by SC RFA, they developed an algorithm that relies on personal identifying information allowing linkage between the maternal and offspring databases [10]. Both maternal and offspring IDs were assigned across databases. Linked birth certificate, inpatient, ED and Medicaid eligibility files were available from 2020 through 2022. Of the 144,272 pregnancies that resulted in a live singleton birth, maternal inpatient hospital procedure and diagnostic codes from delivery were successfully linked for 141,367 (97.99%) deliveries, while infant hospital procedure and diagnostic codes from delivery were successfully linked for 139,824 (96.92%) of births. The Institutional Review Board approved the study as exempt research.

### 2.2 Definitions

Table S1 displays definitions and the International Classification of Diseases, Tenth Revision, Clinical Modification (ICD‐10‐CM) diagnostic codes from hospitalization/ED visit, birth certificate, and death certificate data for the exposure, outcomes, and covariates. SC DHEC data for all reported cases were used to define COVID‐19 diagnoses. The date of the reported severe acute respiratory syndrome coronavirus 2 (SARS‐CoV‐2) diagnostic test and gestational age at diagnosis were used to categorize positive COVID‐19 diagnoses based on earliest exposure occurring before pregnancy, during the first/second trimester (up to 27 6/7 weeks of gestation), or during the third trimester (28 0/7 weeks until delivery). Unexposed pregnancies were defined as those with no record of COVID‐19 before or during pregnancy. Maternal and perinatal outcomes included hypertensive disorders of pregnancy (HDP; gestational hypertension, preeclampsia, eclampsia), preeclampsia and eclampsia, PTD ≤ 37 0/7 weeks, placental abruption, postpartum hemorrhage, severe maternal morbidity (SMM) based on the Centers for Disease Control and Prevention (CDC) definition [11] with and without transfusion, GDM, C‐section, and stillbirth (gestational age of ≥ 20 weeks) (Table S1). Infant outcomes included small for gestational age (SGA), NICU admission, and infant mortality. Maternal records were available at least 3 years before each pregnancy.

### 2.3 Covariates

Calendar time at delivery was defined in quarters. Maternal age was defined as reported on the birth certificate. Maternal race and ethnicity were self‐identified as non‐Hispanic Black (NHB), non‐Hispanic White (NHW), Hispanic/Latina, and other race/ethnicity (including Asian, American Indian and Other) and defined based on the most common report across data sources with the caveat that pregnant women who self‐identified as Hispanic/Latina ethnicity three or more times were classified as Hispanic/Latina. Medicaid eligibility within two months of delivery was available from a statewide Medicaid eligibility file. Variables reported on the birth certificate included education, rural versus urban residence, smoking during or pre‐pregnancy, and previous PTD. Birth certificate‐reported height and weight were used to calculate pre‐pregnancy body mass index (BMI; kg/m^2^) classified as underweight (14.0–18.4), normal (18.5–24.9), overweight (25.0–29.9), or obese (≥30). Clinical covariates included pre‐pregnancy hypertension and pre‐pregnancy diabetes (Table S1). Firstborn included the first live or stillborn infant reported on the birth certificate.

### 2.4 Statistical analysis

The overarching objective of this study is to examine the association between maternal COVID‐19 infection, its timing based on pregnancy onset and the trimester of exposure, and pregnancy and infant outcomes. To achieve this, we used t‐tests and chi‐square tests to examine the association between sociodemographic, lifestyle, clinical characteristics, and maternal complications, and COVID‐19 exposure. Next, we fitted log‐binomial models—i.e., binomial generalized linear models that use a log link—to estimate risk ratios (RRs) for maternal and infant outcomes in unmatched and matched analyses. However, if log‐binomial models failed to converge, we instead used Poisson regressions with sandwich standard errors (i.e., modified Poisson model) to obtain robust variance estimates [12].

Two datasets were used for the unmatched analysis. The first included all pregnancies and examined outcomes, comparing those with COVID‐19 pre‐pregnancy and COVID‐19 during the first or second trimester to those with no history of COVID‐19. The second was limited to pregnancies that entered the third trimester, comparing those with COVID‐19 during the third trimester to those with no history of COVID‐19. Figure S1 shows the consort diagram.

For the matched analysis, women in the non‐exposed group were 1:1 exact matched to those in the COVID‐19 exposure group by race‐ethnicity, delivery quarter, and Medicaid eligibility. Additionally, maternal age was propensity‐score matched using the nearest neighbor method, with 99% of pairs being exact matched [13]. Each COVID‐19 exposure timing group was matched to a non‐exposed control group, resulting in three matched analyses corresponding to COVID‐19 diagnoses occurring (1) pre‐pregnancy, (2) during the first and/or second trimester, and (3) during the third trimester. The non‐exposed control group matched to COVID‐19 exposure during the third trimester was limited to pregnancies that entered the third trimester.

Covariates were identified a priori. For the matched analysis, five imputed datasets were sufficient to maintain model stability due to the low percentage of missing data (2%) [14]. Log‐binomial and modified Poisson models were fitted in each imputed dataset, and the pooled estimates and standard errors across imputed datasets were calculated using Rubin's rules [15, 16]. Models fitted for each outcome in the three matched datasets included COVID‐19 exposure and sociodemographic variables (delivery quarter time, maternal age, Medicaid eligibility, race‐ethnicity, education, and rural residence) in Model 1, and Model 1 with further adjustment for lifestyle (smoking during or pre‐pregnancy) and clinical variables (firstborn, previous PTD, pre‐pregnancy hypertension, pre‐pregnancy diabetes, and pre‐pregnancy BMI), depending on the outcome of interest in Model 2. *p* values of 0.05 and corresponding 95% CIs were used to determine statistical significance. All analyses were performed using R software (R Core Team, 2021) and SAS version 9.4 (SAS Institute, Cary, NC, USA).

## 3 RESULTS

During the study period, there were 145,028 pregnancies that resulted in a fetal death (*n* = 756) or live singleton birth (*n* = 144,272). Of these pregnancies, 7.5% (*n* = 10,942) were first exposed to COVID‐19 before pregnancy, with 5.5% (*n* = 7955) exposed during the first or second trimester, and 3.4% (*n* = 4945) exposed during the third trimester. Of the pregnancies exposed to COVID‐19 prior to pregnancy, COVID‐19 exposures occurred a median of 5.7 months before pregnancy (the 25th to 75th percentiles for COVID‐19 onset ranged from 2.4–10.3 months). Table 1 presents maternal and infant characteristics and outcomes for the COVID‐19 pre‐pregnancy and first/second‐trimester exposure groups, their respective matched control groups, and the overall population of 140,077 pregnancies. The mean age at delivery was 28.4 years and 58.0% of pregnancies were Medicaid‐eligible. Characteristics of the COVID‐19 third trimester exposure group, its matched control group, and the overall population of 125,091 pregnancies that entered the third trimester are found in Table S2.

**TABLE 1:** Mean, standard deviation, and percent for sociodemographic and clinical characteristics of deliveries in South Carolina by COVID‐19 infection status and timing, 2020–2022, for matched datasets 1 and 2.^a^

	COVID‐19 infection				Overall (unmatched)^b^
	Matched analysis 1^a^, ^b^, ^*^		Matched analysis 2^a^, ^b^, ^*^		
	No COVID‐19	COVID‐19 pre‐pregnancy	No COVID‐19	Covid‐19 1st/2nd trimester	
Characteristic, *n* (%)	*n* = 10,942	*n* = 10,942	*n* = 7955	*n* = 7955	*n* = 140,077
**Covariates**					
Mothers age at delivery; mean (SD)	28.5 (5.6)	28.5 (5.6)	28.4 (5.59)	28.4 (5.59)	28.4 (5.8)
Medicaid	6675 (61.0)	6675 (61.0)	4660 (58.6)	4660 (58.6)	81,272 (58.0)
Mother's race‐ethnicity					
Non‐Hispanic White	5941 (54.3)	5941 (54.3)	4479 (56.3)	4479 (56.3)	78,230 (55.9)
Non‐Hispanic Black	3789 (34.6)	3789 (34.6)	2665 (33.5)	2665 (33.5)	42,161 (30.1)
Hispanic	811 (7.4)	811 (7.4)	500 (6.3)	500 (6.3)	12,758 (9.1)
Other race‐ethnicity	401 (3.7)	401 (3.7)	311 (3.9)	311 (3.9)	6928 (5.0)
Mother's education					
Less than high school	1259 (11.6)	882 (8.1)	883 (11.1)	675 (8.5)	16,920 (12.1)
High school	3214 (29.5)	2946 (27.0)	2264 (28.6)	2110 (26.6)	36,931 (26.4)
Some college	2412 (22.1)	2676 (24.5)	1752 (22.1)	1952 (24.6)	31,436 (22.4)
College graduate	4011 (36.8)	4410 (40.4)	3026 (38.2)	3191 (40.2)	54,251 (38.7)
Rural residence	3533 (32.3)	3537 (32.3)	2489 (31.3)	2507 (31.5)	42,609 (30.4)
Smoking (during or pre‐pregnancy)	952 (8.7)	524 (4.8)	682 (8.6)	520 (6.5)	11,458 (8.2)
Firstborn	3335 (30.5)	3335 (30.5)	2449 (30.8)	2416 (30.4)	44,373 (31.7)
Previous preterm birth	643 (5.9)	642 (5.9)	432 (5.4)	457 (5.7)	7299 (5.2)
Pre‐pregnancy hypertension	1366 (12.5)	1648 (15.1)	965 (12.1)	1056 (13.3)	15,502 (11.1)
Pre‐pregnancy diabetes	303 (2.8)	402 (3.7)	220 (2.8)	235 (3.0)	3426 (2.5)
Pre‐pregnancy BMI category					
Underweight	361 (3.3)	260 (2.4)	306 (3.9)	198 (2.5)	4151 (3.0)
Normal	3817 (35.1)	3318 (30.5)	2753 (34.8)	2520 (31.9)	49,741 (35.5)
Overweight	2693 (24.8)	2779 (25.5)	1939 (24.5)	2007 (25.4)	35,911 (25.6)
Obese	3997 (36.8)	4525 (41.6)	2903 (36.7)	3169 (40.1)	48,807 (34.8)
**Maternal outcomes**					
C‐section	3428 (31.3)	3709 (33.9)	2482 (31.2)	2574 (32.4)	44,549 (31.8)
Fetal death	51 (0.5)	66 (0.6)	33 (0.4)	42 (0.5)	734 (0.5)
GDM	1214 (11.1)	1196 (10.9)	845 (10.6)	839 (10.5)	14,406 (10.3)
HDP	2141 (19.6)	2449 (22.4)	1487 (18.7)	1618 (20.3)	25,927 (18.5)
Placental abruption	632 (5.8)	635 (5.8)	404 (5.1)	467 (5.9)	7430 (5.3)
Postpartum hemorrhage	523 (4.8)	510 (4.7)	340 (4.3)	380 (4.8)	6004 (4.3)
Preeclampsia & eclampsia	1065 (9.7)	1242 (11.4)	747 (9.4)	845 (10.6)	12,798 (9.1)
Preterm delivery	1210 (11.1)	1322 (12.1)	844 (10.6)	889 (11.2)	14,585 (10.4)
SMM without blood transfusion	159 (1.5)	192 (1.8)	109 (1.4)	209 (2.6)	2032 (1.5)
SMM with blood transfusion	240 (2.2)	285 (2.6)	168 (2.1)	270 (3.4)	3250 (2.3)
**Infant outcomes**					
Infant mortality	54 (0.5)	67 (0.6)	47 (0.6)	43 (0.5)	753 (0.5)
NICU admission	972 (8.9)	1027 (9.4)	675 (8.5)	710 (9.0)	11,499 (8.2)
SGA	1257 (11.6)	1073 (9.9)	835 (10.6)	715 (9.1)	14,127 (10.1)

Abbreviations: BMI, body mass index; C‐section, Cesarean birth; GDM, gestational diabetes mellitus; HDP, hypertensive disorders of pregnancy; NICU, neonatal intensive care unit; SD, standard deviation; SGA, small for gestational age; SMM, severe maternal morbidity.

^a^ The COVID‐19 exposure group and the non‐exposed group are matched by maternal age at delivery, race‐ethnicity group, Medicaid eligibility, and delivery quarter time. The other race‐ethnicity group included pregnancies from those who identified as Asian (28.3%), American Indian (3.6%), and other race‐ethnicity (68.1%).

^b^ Number of missing values (Overall): education (539), smoking (52), firstborn (152), BMI category (1467), preterm delivery (54), SGA (994), NICU admission (734). Number of missing values (Analysis I): education (74), smoking (10), firstborn (46), BMI category (134), preterm delivery (11), SGA (168), NICU admission (117). Number of missing values (Analysis II): education (57), smoking (2), firstborn (25), BMI category (115), preterm delivery (8), SGA (110), NICU admission (75).

* *p* values (Analysis 1): education, smoking, pre‐pregnancy hypertension, pre‐pregnancy diabetes, pre‐pregnancy BMI category, C‐section, HDP, preeclampsia & eclampsia, and SGA (< 0.001) and preterm delivery (0.02). *p* values (Analysis 2): education, smoking, pre‐pregnancy hypertension, pre‐pregnancy BMI category, SMM without blood transfusion, and SMM with blood transfusion (< 0.001); HDP (0.009); placental abruption (0.03); preeclampsia & eclampsia (0.01); SGA (0.002).

In our unmatched analysis adjusted for sociodemographic characteristics, lifestyle, and clinical factors, COVID‐19 infection before pregnancy relative to no history of COVID‐19 infection increased the risk of HDP (RR = 1.06, 95% CI: 1.02–1.10), preeclampsia/eclampsia (RR = 1.10, 95% CI: 1.03–1.16), PTD (RR = 1.12, 95% CI: 1.06–1.19), and C‐section (RR = 1.06, 95% CI: 1.02–1.10) (Table 2: Unmatched Model 2). In a parallel matched analysis, COVID‐19 infection before pregnancy increased the risk for HDP (RR = 1.07, 95% CI: 1.03–1.14), preeclampsia/eclampsia (RR = 1.09, 95% CI: 1.01–1.17), and C‐section (RR = 1.06, 95% CI: 1.00–1.11) (Table 2: Matched Analyses 1 Model 2).

**TABLE 2:** Association between first COVID‐19 diagnosis pre‐pregnancy or during the 1st and/or 2nd trimester with adverse maternal outcomes, 2020–2022.

	Unmatched analysis		Matched an alysis 1:^a^ pre‐pregnancy		Matched analysis 2:^a^ 1st/2nd trimester	
Maternal outcomes	Model 1^b^	Model 2^c^	Model 1^b^	Model 2^c^	Model 1^b^	Model 2^c^
HDP						
No history of COVID‐19	REF	REF	REF	REF	REF	REF
COVID‐19 pre‐pregnancy	**1.15 (1.11, 1.20)**	**1.06 (1.02, 1.10)**	**1.14 (1.08, 1.20)**	**1.07 (1.03, 1.14)** ^d^	—	—
COVID‐19 1st/2nd trimester	**1.07 (1.03, 1.13)**	1.03 (0.99, 1.08)	—	—	**1.09 (1.03, 1.16)**	1.05 (0.99, 1.12)^d^
Preeclampsia & eclampsia						
No history of COVID‐19	REF	REF	REF	REF	REF	REF
COVID‐19 pre‐pregnancy	**1.21 (1.14, 1.28)**	**1.10 (1.03, 1.16)** ^d^	**1.17 (1.08, 1.26)**	**1.09 (1.01, 1.17)** ^d^	—	—
COVID‐19 1st/2nd trimester	**1.15 (1.08, 1.23)**	**1.10 (1.03, 1.18)** ^d^	—	—	**1.14 (1.03, 1.25)**	**1.10 (1.00, 1.20)** ^d^
Preterm delivery						
No history of COVID‐19	REF	REF	REF	REF	REF	REF
COVID‐19 pre‐pregnancy	**1.17 (1.11, 1.24)**	**1.12 (1.06, 1.19)** ^d^	**1.10 (1.02, 1.18)**	1.07 (0.99, 1.15)^d^	—	—
COVID‐19 1st/2nd trimester	**1.09 (1.02, 1.17)**	1.07 (1.00, 1.14)^d^	—	—	1.06 (0.97, 1.16)	1.04 (0.95, 1.13)^d^
Placental abruption						
No history of COVID‐19	REF	REF	REF	REF	REF	REF
COVID‐19 pre‐pregnancy	1.06 (0.98, 1.15)	1.05 (0.97, 1.15)	1.01 (0.91, 1.13)	1.00 (0.90, 1.11)^d^	—	—
COVID‐19 1st/2nd trimester	**1.10 (1.01, 1.21)**	**1.11 (1.01, 1.21)**	—	—	**1.15 (1.01, 1.31)**	**1.15 (1.01, 1.30)**
Postpartum hemorrhage						
No history of COVID‐19	REF	REF	REF	REF	REF	REF
COVID‐19 pre‐pregnancy	1.04 (0.94, 1.14)	1.03 (0.93, 1.13)	0.98 (0.87, 1.04)	0.96 (0.85, 1.08)	—	—
COVID‐19 1st/2nd trimester	1.10 (0.99, 1.22)	1.10 (0.99, 1.22)	—	—	1.11 (0.96, 1.28)	1.10 (0.95, 1.27)
SMM without transfusion						
No history of COVID‐19	REF	REF	REF	REF	REF	REF
COVID‐19 pre‐pregnancy	**1.18 (1.00, 1.38)** ^d^	1.09 (0.92, 1.28)^d^	**1.23 (1.00, 1.52)**	1.16 (0.94, 1.43)	—	—
COVID‐19 1st/2nd trimester	**1.84 (1.59, 2.13)** ^d^	**1.76 (1.51, 2.04)** ^d^	—	—	**1.97 (1.57, 2.48)**	**1.91 (1.52, 2.40)**
SMM with transfusion						
No history of COVID‐19	REF	REF	REF	REF	REF	REF
COVID‐19 pre‐pregnancy	1.11 (0.98, 1.26)	1.06 (0.93, 1.21)^d^	**1.20 (1.02, 1.43)**	1.16 (0.98, 1.38)	—	—
COVID‐19 1st/2nd trimester	**1.48 (1.30, 1.68)**	**1.44 (1.27, 1.64)** ^d^	—	—	**1.64 (1.36, 1.98)**	**1.60 (1.32, 1.93)**
GDM						
No history of COVID‐19	REF	REF	REF	REF	REF	REF
COVID‐19 pre‐pregnancy	**1.06 (1.00, 1.12)** ^d^	0.98 (0.92, 1.04)^d^	1.00 (0.92, 1.07)	0.94 (0.87, 1.01)^d^	—	—
COVID‐19 1st/2nd trimester	1.03 (0.97, 1.10)^d^	0.98 (0.92, 1.04)^d^	—	—	0.99 (0.90, 1.08)	0.95 (0.87, 1.04)^d^
Cesarean delivery						
No history of COVID‐19	REF	REF	REF	REF	REF	REF
COVID‐19 pre‐pregnancy	**1.10 (1.06, 1.15)**	**1.06 (1.02, 1.10)** ^d^	**1.10 (1.04, 1.16)**	**1.06 (1.00, 1.11)** ^d^	—	—
COVID‐19 1st/2nd trimester	1.02 (0.97, 1.07)	1.01 (0.96, 1.05)^d^	—	—	1.04 (0.98, 1.11)	1.04 (0.97, 1.10)^d^
Fetal death						
No history of COVID‐19	REF	REF	REF	REF	REF	REF
COVID‐19 pre‐pregnancy	1.21 (0.91, 1.60)	1.17 (0.83, 1.65)	1.33 (0.92, 1.91)	1.30 (0.90, 1.87)	—	—
COVID‐19 1st/2nd trimester	1.13 (0.82, 1.55)	1.19 (0.83, 1.70)	—	—	1.30 (0.83, 2.06)	1.29 (0.82, 2.04)

Abbreviations: CI, confidence interval; C‐section, Cesarean delivery; GDM, gestational diabetes mellitus; HDP, hypertensive disorders or pregnancy; RR, risk ratio; SMM, severe maternal morbidity.

^a^ The COVID‐19 exposure group and non‐exposed group are matched by maternal age at delivery, race‐ethnicity group, Medicaid eligibility, and delivery quarter time.

^b^ Model 1 is adjusted for maternal age, delivery quarter time, Medicaid, race‐ethnicity, education, and rural residence.

^c^ Model 2 is additionally adjusted for smoking during or pre‐pregnancy, firstborn, previous preterm delivery, pre‐pregnancy hypertension, pre‐pregnancy diabetes, and pre‐pregnancy BMI.

^d^ A modified Poisson model was used due to the convergence issues with the log‐binomial model.

In our unmatched analysis compared with pregnant women without COVID‐19 during the first or second trimester, those with COVID‐19 in the same trimesters had elevated risk of preeclampsia/ eclampsia (RR = 1.10, 95% CI: 1.03–1.18), placental abruption (RR = 1.11, 95% CI: 1.01–1.21), SMM without transfusion (RR = 1.76, 95% CI: 1.51–2.04), and SMM with transfusion (RR = 1.44, 95% CI: 1.27–1.64) (Table 2: Unmatched Model 2). In a parallel matched analysis, COVID‐19 infection during the first or second trimester increased the risk for preeclampsia/eclampsia (RR = 1.10, 95% CI 1.00–1.20), placental abruption (RR = 1.15, 95% CI: 1.01–1.30), SMM without transfusion (RR = 1.91, 95% CI: 1.52–2.40), and SMM with transfusion (RR = 1.60, 95% CI: 1.32–1.93) (Table 2: Matched Analyses 2 Model 2).

Among pregnancies that entered the third trimester relative to no history of COVID‐19 infection, COVID‐19 during the third trimester elevated the risk of preeclampsia/eclampsia (RR = 1.10, 95% CI: 1.01–1.20), SMM without transfusion (RR = 2.64, 95% CI: 2.26–3.09), and SMM with transfusion (RR = 2.03, 95% CI: 1.78–2.33) (Table 3: Unmatched Model 2). In a matched analysis, COVID‐19 infection remained significantly associated with preeclampsia/eclampsia (RR = 1.15, 95% CI: 1.02–1.29), placenta abruption (RR = 1.38, 95% CI: 1.15–1.66), postpartum hemorrhage (RR = 1.24, 95% CI: 1.02–1.51), SMM without transfusion (RR = 2.56, 95% CI: 1.98–3.35), and SMM with transfusion (RR = 2.07, 95% CI: 1.65–2.59) (Table 3: Matched Analyses 3 Model 2).

**TABLE 3:** Association between first COVID‐19 diagnosis during the 3rd trimester (≥ 28 weeks gestational age) with adverse maternal outcomes, 2020–2022.^a^

	Unmatched analysis		Matched analysis 3: 3rd trimester^b^	
Maternal outcomes	Model 1^c^	Model 2^d^	Model 1^c^	Model 2^d^
HDP				
No history of COVID‐19	REF	REF	REF	REF
COVID‐19 during 3rd trimester	**1.06 (1.00, 1.13)**	1.04 (0.99, 1.10)	1.06 (0.98, 1.15)	1.04 (0.96, 1.13)^e^
Preeclampsia & eclampsia				
No history of COVID‐19	REF	REF	REF	REF
COVID‐19 during 3rd trimester	**1.12 (1.03, 1.22)**	**1.10 (1.01, 1.20)** ^e^	**1.16 (1.02, 1.31)**	**1.15 (1.02, 1.29)** ^e^
Preterm delivery				
No history of COVID‐19	REF	REF	REF	REF
COVID‐19 during 3rd trimester	0.98 (0.90, 1.08)	0.98 (0.90, 1.07)^e^	1.02 (0.90, 1.15)	1.02 (0.91, 1.15)^e^
Placental abruption				
No history of COVID‐19	REF	REF	REF	REF
COVID‐19 during 3rd trimester	1.04 (0.92, 1.18)	1.06 (0.94, 1.20)	**1.38 (1.15, 1.66)**	**1.38 (1.15, 1.66)**
Postpartum hemorrhage				
No history of COVID‐19	REF	REF	REF	REF
COVID‐19 during 3rd trimester	1.02 (0.89, 1.17)	1.04 (0.90, 1.19)	**1.24 (1.02, 1.51)**	**1.24 (1.02, 1.51)**
SMM without transfusion				
No history of COVID‐19	REF	REF	REF	REF
COVID‐19 during 3rd trimester	**2.65 (2.28, 3.10)** ^e^	**2.64 (2.26, 3.09)** ^e^	**2.61 (1.99, 3.42)**	**2.56 (1.98, 3.35)**
SMM with transfusion				
No history of COVID‐19	REF	REF	REF	REF
COVID‐19 during 3rd trimester	**2.02 (1.76, 2.30)**	**2.03 (1.78, 2.33)** ^e^	**2.10 (1.68, 2.62)**	**2.07 (1.65, 2.59)**
Fetal death				
No history of COVID‐19	REF	REF	REF	REF
COVID‐19 during 3rd trimester	1.30 (0.83, 2.01)	1.38 (0.86, 2.22)	1.70 (0.86, 3.37)	1.66 (0.84, 3.29)
GDM				
No history of COVID‐19	REF	REF	REF	REF
COVID‐19 during 3rd trimester	1.05 (0.97, 1.14)^e^	1.01 (0.94, 1.10)^e^	1.03 (0.91, 1.15)	0.99 (0.89, 1.11)^e^
Cesarean delivery				
No history of COVID‐19	REF	REF	REF	REF
COVID‐19 during 3rd trimester	1.02 (0.96, 1.08)	1.01 (0.95, 1.07)^e^	1.00 (0.93, 1.09)	1.01 (0.93, 1.09)^e^

Abbreviations: CI, confidence interval; C‐section, Cesarean delivery; GDM, gestational diabetes mellitus; HDP, hypertensive disorders or pregnancy; RR, risk ratio; SMM, severe maternal morbidity.

Bold values indicate statistical signifcance.

^a^ Pregnancies with first diagnosis of COVID‐19 during the 1st and/or 2nd trimester were excluded.

^b^ The COVID‐19 exposure group and the non‐exposed group are matched by maternal age at delivery, race‐ethnicity group, Medicaid eligibility, and delivery quarter time.

^c^ Model 1 is adjusted for maternal age, delivery quarter time, Medicaid, race‐ethnicity, education, and rural residence.

^d^ Model 2 is additionally adjusted for smoking during or pre‐pregnancy, firstborn, previous preterm delivery, pre‐pregnancy hypertension, pre‐pregnancy diabetes, and pre‐pregnancy BMI.

^e^ A modified Poisson model was used due to the convergence issues with the log‐binomial model.

Of the three infant outcomes examined in separate matched analyses that adjusted for sociodemographic characteristics, lifestyle, and clinical factors, the only significant associations found were a decreased risk of being SGA for those with COVID‐19 prior to pregnancy (RR = 0.90, 95% CI: 0.83–0.97) and during the first or second trimester (RR = 0.91, 95% CI: 0.82–0.99) (Table S3: Matched Analyses 1 and 2: Models 2) relative to counterparts with no history of COVID‐19. Additionally, in the matched analysis comparing those with COVID‐19 during the third trimester to those without a history of COVID‐19, we report increased risk of NICU admission (RR = 1.16, 95% CI: 1.01–1.33) after adjustment for sociodemographic, lifestyle, and clinical factors that became non‐significant with additional adjustment for gestational age of delivery (RR = 1.13, 95% CI: 0.99–1.29) (Table S4: Matched Analyses 3: Models 2 and 3).

Forest plots for preeclampsia/eclampsia (Figure 1) and SMM without transfusion (Figure 2) are based on Model 2 of Matched Analyses 1, 2, and 3 for COVID‐19 infection occurring prior to pregnancy, during the 1st/2nd semester, or during the 3rd semester, respectively.

FIGURE 1Forest plot of the risk ratios (95% CI) for preeclampsia and eclampsia by COVID‐19 infection status and timing in relation to pregnancy compared to those without COVID‐19: (a) pre‐pregnancy, (b) during the 1st and/or 2nd trimester, and (c) during the 3rd trimester. BMI, body mass index; NHW, non‐Hispanic White.

**Figure:**
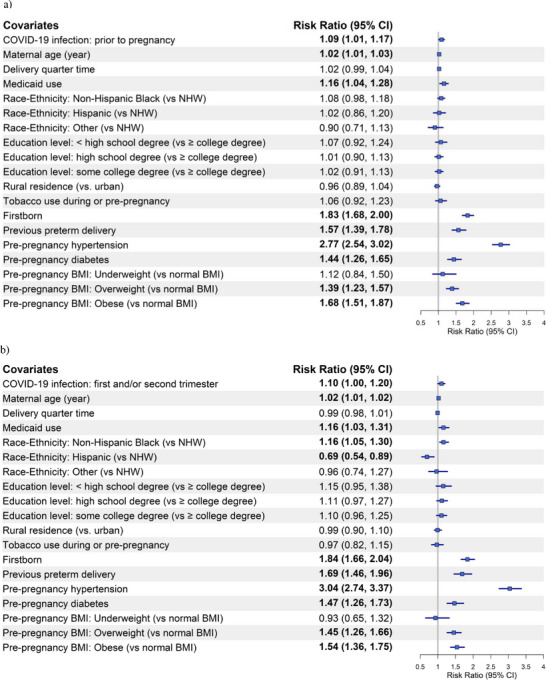

**Figure:**
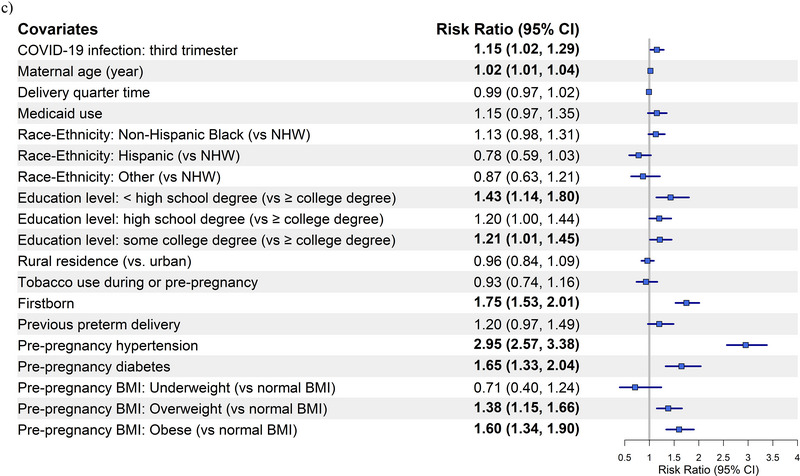

FIGURE 2Forest plot of the risk ratios (95% CI) for severe maternal morbidity (SMM) without transfusion by COVID‐19 infection status and timing in relation to pregnancy compared to those without COVID‐19: (a) pre‐pregnancy, (b) during the 1st and/or 2nd trimester, and (c) during the 3rd trimester. BMI, body mass index; NHW, non‐Hispanic White.

**Figure:**
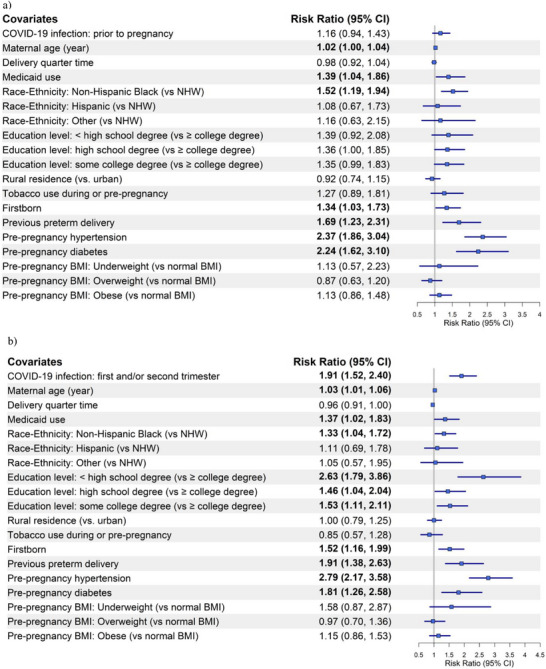

**Figure:**
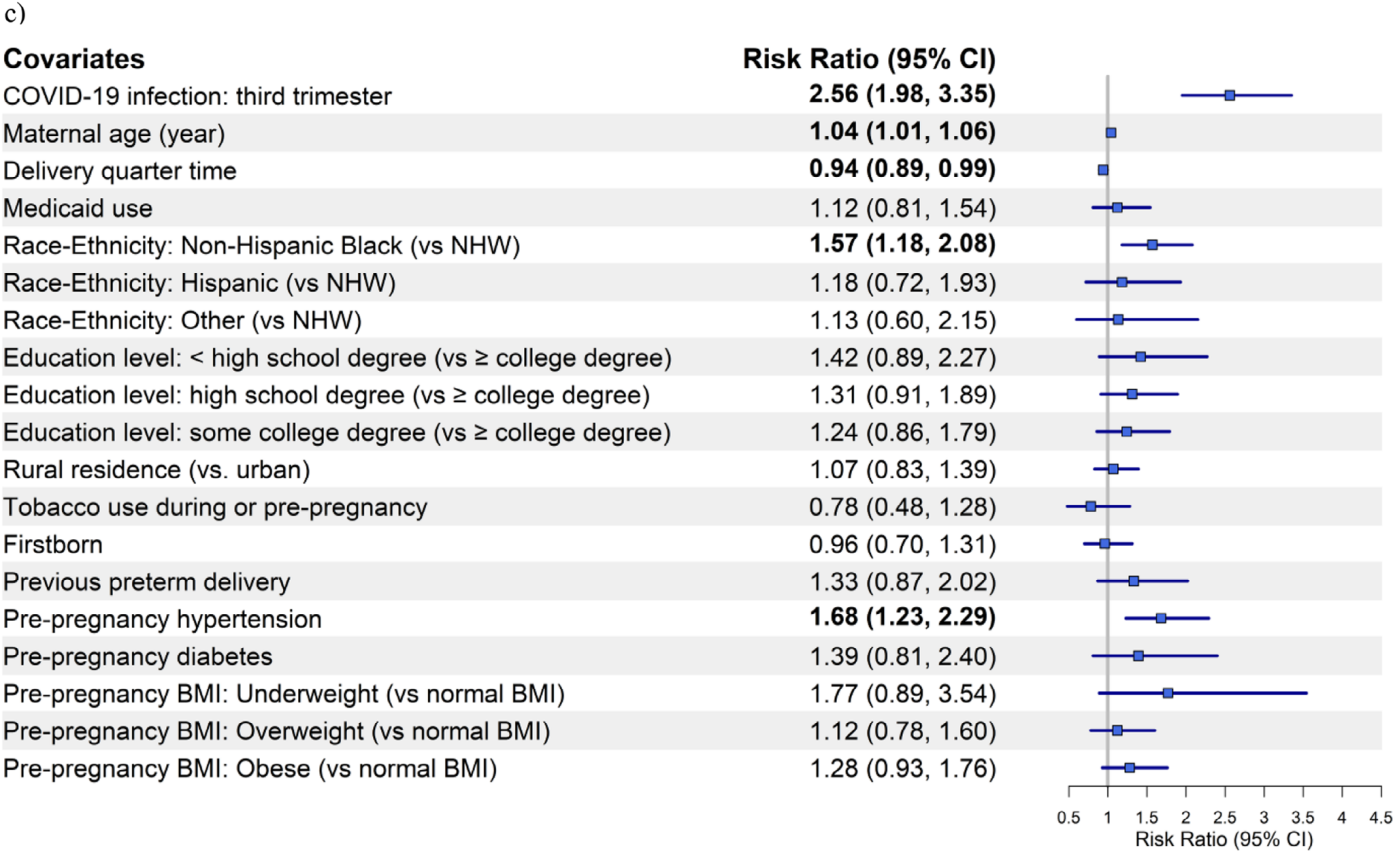

## 4 DISCUSSION

We found significantly increased risk of HDP, specifically preeclampsia/eclampsia, among pregnant women who had COVID‐19 prior to pregnancy compared to those without COVID‐19 infection in a statewide matched analyses and after adjustment for covariates. We also report an increased risk of C‐section associated with having COVID‐19 prior to pregnancy. COVID‐19 infection during the first or second trimester was associated with increased risk for preeclampsia/eclampsia, placental abruption, and SMM (both including and excluding transfusion) relative to those with no history of COVID‐19. Finally, COVID‐19 infection during the third trimester was associated with increased risk of preeclampsia/eclampsia, placental abruption, postpartum hemorrhage, and SMM (both including and excluding transfusion).

Disease onset has been examined in some previous studies, including meta‐analyses that qualified COVID‐19 infection as occurring anytime throughout pregnancy [2, 3, 17, 18, 19], with somewhat inconsistent findings reported when examining COVID‐19 infection timing by trimester of gestation. In addition, our study differs from three recent meta‐analyses of COVID‐19 infection and adverse pregnancy outcomes in that we considered exposure during and prior to pregnancy with no meta‐analyses differentiating between trimester of COVID‐19 exposure [2, 3, 5]. Only one study to our knowledge assessed the effect of COVID‐19 infection prior to pregnancy on pregnancy‐related outcomes [4]. In this small retrospective cohort study (*n* = 71), women with infection pre‐conception had lower birth weights and gestational ages at delivery, and were more likely to experience pregnancy loss before 20 weeks than those without COVID‐19 [4]. They also observed that women with COVID‐19 infection pre‐conception and in the first trimester were especially likely to experience placental vascular changes and accelerated villous maturation.

Thus, while prior studies have examined potential risks associated with COVID‐19 infection during pregnancy, our study is one of the first to investigate potential risks associated with COVID‐19 prior to pregnancy. Our findings of associations between COVID‐19 and increased risk of HDP and preeclampsia/eclampsia, and decreased risk of SGA differ from a recent international meta‐analysis, which did not find an association with HDP or intrauterine growth retardation/SGA [2]. However, the study did report an association between COVID‐19 during pregnancy and an increased risk of PTD, maternal mortality, NICU admission, and neonatal death [2]. In our matched analyses, we reported an association between COVID‐19 exposure during the third trimester and postpartum hemorrhage and NICU admission. A relationship was also observed between pre‐pregnancy COVID‐19 and PTD after initial adjustment that was no longer significant after full adjustment, although we did not replicate the other associations. A second recent international meta‐analysis reported women with COVID‐19 during pregnancy had higher odds of preeclampsia (odds ratio [OR] = 1.41, 95% CI: 1.30–1.53) and placental abruption (OR = 1.40, 95% CI: 1.02–1.92) similar to our findings although they did not replicate the association with postpartum hemorrhage (OR = 0.98; 95% CI, 0.78–1.24) [3]. This meta‐analysis also reported associations for PTD (OR = 1.59, 95% CI: 1.42–1.78), GDM (OR = 1.13, 95% CI: 1.04–1.23), C‐section (OR = 1.20, 95% CI: 1.10–1.30), NICU admission (OR = 2.33, 95% CI: 1.72–3.16), and low birth weight (OR = 1.52, 95% CI: 1.30–1.79) with COVID‐19 infection in pregnancy, whereas intrauterine growth retardation/SGA (OR = 1.12, 95% CI: 1.00–1.26) was not associated [3]. A third international meta‐analysis (2021) observed associations between COVID‐19 during pregnancy and preeclampsia (OR = 1.33, 95% CI: 1.03–1.73), stillbirth (OR = 2.11, 95% CI: 1.14–3.90), and PTD (OR = 1.82, 95% CI: 1.38–2.39), with severe infection (vs. mild) strongly associated with preeclampsia (OR = 4.16, 95% CI: 1.55–11.15), GDM (OR = 1.99, 95% CI: 1.09–3.64), PTD (OR = 4.29, 95% CI: 2.41–7.63), and low birth weight (OR = 1.89, 95% CI: 1.14–3.12) [5].

In the 2020 U.S. National Inpatient Sample, women with COVID‐19 infection in the second trimester experienced lower odds of preeclampsia (aOR = 0.4, 95% CI: 0.2–0.61) and higher odds of GDM (aOR = 1.4, 95% CI: 1.01–1.9) than those without COVID‐19; no relationships were observed for gestational hypertension or preterm labor after potential confounder adjustment [20]. COVID‐19 infection in the third trimester increased the odds of preeclampsia (aOR = 1.17, 95% CI: 1.1–1.3) as well as preterm labor (aOR = 1.3, 95% CI: 1.1–1.6) although was not associated with GDM, gestational hypertension, or eclampsia after adjustment [20]. A retrospective cohort study of women who delivered at Kaiser Permanente hospitals in Southern California did not observe an association between COVID‐19 infection during pregnancy and preeclampsia/eclampsia (adjusted odds ratio [aOR] = 1.14, 95% CI: 0.98–1.33), although the odds of spontaneous PTD were increased (aOR = 1.28, 95% CI: 1.03–1.58) and higher for earlier infections [21]. Another retrospective cohort study of 17 hospitals in the U.S. States observed an association between COVID‐19 infection in the first or second trimester with PTD (adjusted relative risk [aRR] = 1.29, 95% CI: 1.02–1.63) and HDP (aRR = 1.74, 95% CI: 1.19–2.55) at < 37 weeks of gestation, as well as with fetal or neonatal death (aRR = 1.97, 95% CI: 1.01–3.85), although no association was observed for preeclampsia or gestational hypertension overall [22]. Increased risk of PTD was also found among pregnant women with COVID‐19 infection mainly in the first and second trimester compared to those without infection by a U.S. retrospective cohort study [19]. While we found an increased risk for PTD among women with COVID‐19 prior to pregnancy compared to those without after adjustment in Model 1 (RR = 1.10, 95% CI: 1.02–1.18), the relationship attenuated and was no longer statistically significant in the fully adjusted Model 2 (RR = 1.07, 95% CI: 0.99, 1.15).

We reported associations with COVID‐19 for increased risk of placental abruption (first, second, and third trimester), postpartum hemorrhage (third trimester), and NICU (third trimester). In contrast, we report a decreased risk of SGA associated with COVID‐19 exposure during the first or second, but not the third trimester. This is not consistent with prior findings including a 2023 systematic review and meta‐analysis that did not observe a relationship between pregnant women or women attempting to become pregnant with current or past COVID‐19 infection and SGA; however, timing by trimester was not known [23]. Similarly, COVID‐19 infection overall and by trimester was not associated with SGA in a population‐based study in Israel [24], nor was an association found in a Mexican retrospective cohort study [25]. We do not have a plausible biologic explanation for our findings of decreased risk of SGA and increased risk of HDP which may be a spurious finding.

Our study also found significantly increased risk of experiencing SMM with or without transfusion among those with COVID‐19 infection in the first/second trimester or the third trimester of pregnancy compared to those without COVID‐19. While we defined SMM according to the CDC's indicators, it is important to consider the variety of definitions used throughout the existing literature in relation to findings [26]. One international study differed in its SMM definition, yet still observed significant associations with COVID‐19 infection [18]. Moreover, our findings corroborate those published by the University of Southern California using the CDC's definition for SMM [27].

In regard to previous primary studies examining the association between COVID‐19 infection and pregnancy outcomes, ours is the second to our knowledge to consider the impact of infection prior to pregnancy. Some studies defined COVID‐19 infection during pregnancy as contracting the virus anytime throughout pregnancy, while others only considered infection around the time of delivery [3, 28, 29]. More recent publications have typically only included data through 2020 or 2021, while ours includes statewide data for SC from 2020–2022 [3, 27, 28, 29]. Additionally, most studies have been designed as case‐control studies, whereas the current study involves a longitudinal population‐based cohort using a large statewide database with linked birth certificate, hospitalization, and ED visit data. The database also included all reported diagnosed COVID‐19 infections in SC during the timeframe when COVID‐19 was reportable to the health department. Therefore, it included the timing of COVID‐19 infection with respect to calendar time, which further allowed us to account for temporal trends with respect to COVID‐19 type and treatment. Moreover, it was possible to differentiate between pre‐pregnancy (chronic) hypertension and HDP, and the database included individual‐level data on clinical characteristics and smoking. We were able to match patients on a variety of sociodemographic factors when examining COVID‐19 infection and pregnancy outcomes [27, 29, 30].

Our study has some limitations including those typical of administrative database studies. We utilized a statewide database of all reported COVID‐19 infections although were unable to account for undiagnosed or unreported infections. Our data sources did not include information on maternal sleep, diet, physical activity, medication, or levels of stress, blood pressure, and glucose. We were not able to adjust for COVID‐19 vaccination status and enough time had also not elapsed to examine timing of COVID‐19 exposure prior to pregnancy (i.e., periconception, months or years). The potential for miscoding exists for pre‐pregnancy diabetes and GDM, pre‐pregnancy hypertension and HDP, and for self‐identified race and ethnicity viewed as social constructs. Our analysis was also not able to control for the consistency and timing of prenatal care because of the uncertainty of birth certificate data on prenatal care as well as the changes in prenatal care observed before and during the pandemic.

## 5 CONCLUSION

Our findings suggest that consideration of COVID‐19 infection prior to and throughout pregnancy should be emphasized. With the increased risk of adverse events associated with COVID‐19 infection, providers should educate pregnant patients about potential risks, especially patients with relevant comorbidities that could further exacerbate those risks. It is imperative to develop risk prevention strategies that target those most at risk of experiencing an adverse event due to COVID‐19 infection in pregnancy.

In the future, it would be important to consider the proximity COVID‐19 infection in relation to a subsequent pregnancy. Additionally, it would be helpful to include information on vaccination status in the association between COVID‐19 infection and pregnancy outcomes, as one study reported pregnant women who were vaccinated against COVID‐19 had significantly lower risk of SMM and mortality compared to those not vaccinated [18]. While our study was unable to account for vaccination status, we still observed significant increases in the risk of pregnancy complications and adverse outcomes among pregnant women with COVID‐19 infection prior to and throughout pregnancy.

Overall, our study found that COVID‐19 infection during and before pregnancy was significantly associated with multiple adverse pregnancy events, even after controlling for pertinent sociodemographic, clinical, and behavioral factors.

## CONFLICT OF INTEREST STATEMENT

The authors declare no conflicts of interest.

## ETHICS STATEMENT

The study was conducted in accordance with the Declaration of Helsinki and approved by the Institutional Review Board of the Medical University of South Carolina (protocol number Pro00117581, January 20, 2022).

## CONSENT

Patient consent was waived as data in this study were collected from previously existing datasets. MUSC researchers received a final limited identified dataset stripped of most personal identifiers by certified state abstractors.

## Supporting information



Supporting Information

## Data Availability

Restrictions apply to the availability of these data. The data used for this study cannot be shared due to the policies of the South Carolina (SC) Revenue and Fiscal Affairs (RFA) Office, the Health and Demographics Section, and the SC Department of Health and Environmental Control (DHEC).

## References

[1] Centers for Disease Control and Prevention . 2024. COVID Data Tracker: U.S. Department of Health and Human Services CDC. https://covid.cdc.gov/covid‐data‐tracker

[2] Simbar, M. , S. Nazarpour , and A. Sheidaei . 2023. “Evaluation of Pregnancy Outcomes in Mothers with COVID‐19 Infection: A Systematic Review and Meta‐Analysis.” Journal of Obstetrics and Gynaecology 43(1): 2162867. 10.1080/01443615.2022.2162867. 36651606

[3] Jeong, Y. and M. A. Kim . 2023. “The Coronavirus Disease 2019 Infection in Pregnancy and Adverse Pregnancy Outcomes: A Systematic Review and Meta‐Analysis.” Obstetrics & Gynecology Science 66(4): 270–89. 10.5468/ogs.22323 [published Online First: 20230517]. PMC10375217.[CrossRef] 37194243 PMC10375217

[4] Hernandez, P. V. , L. Chen , R. Zhang , R. Jackups , D. M. Nelson , and M. He . 2023. “The Effects of Preconception and Early Gestation SARS‐CoV‐2 Infection on Pregnancy Outcomes and Placental Pathology.” Annals of Diagnostic Pathology 62: 152076. 10.1016/j.anndiagpath.2022.152076 [published Online First: 20221205]. PMC9721196.[CrossRef] 36495735 PMC9721196

[5] Wei, S. Q. , M. Bilodeau‐Bertrand , S. Liu , and N. Auger . 2021. “The Impact of COVID‐19 on Pregnancy Outcomes: A Systematic Review and Meta‐Analysis.” Cmaj 193(16): E540–48. 10.1503/cmaj.202604 [published Online First: 2021/03/21]. PMC8084555.[CrossRef] 33741725 PMC8084555

[6] Xie, Y. , E. Xu , B. Bowe , and Z. Al‐Aly . 2022. “Long‐Term Cardiovascular Outcomes of COVID‐19.” Nature Medicine 28(3): 583–90. 10.1038/s41591-022-01689-3 [published Online First: 20220207]. PMC8938267.[CrossRef] PMC8938267 35132265

[7] Abbasi, J . 2022. “The COVID Heart‐One Year after SARS‐CoV‐2 Infection, Patients Have an Array of Increased Cardiovascular Risks.” Jama 327(12): 1113–4. 10.1001/jama.2022.2411. 35234824

[8] Liuzzo, G. and M. Volpe . 2022. “SARS‐CoV‐2 Infection Markedly Increases Long‐Term Cardiovascular Risk.” European Heart Journal 43(20): 1899–00. 10.1093/eurheartj/ehac168. PMC9383629.[CrossRef] 35362024 PMC9383629

[9] Kole, C. , E. Stefanou , N. Karvelas , D. Schizas , and K. P. Toutouzas . 2024. “Acute and Post‐Acute COVID‐19 Cardiovascular Complications: A Comprehensive Review.” Cardiovascular Drugs and Therapy 38(5): 1017–32. 10.1007/s10557-023-07465-w [published Online First: 20230520]. PMC10199303.[CrossRef] 37209261 PMC10199303

[10] Public Health Informatics Institute . 2023. “Child and Adolescent Mental Health.” Integrated data system fosters collaboration across public agencies in South Carolina: Public Health Informatics Institute. The Task Force for Global Health; [cited 2025 April 21]. Available from: https://phii.org/camh‐resources/camh‐user‐stories/south‐carolina‐camh‐user‐story/; https://phii.org/wp‐content/uploads/2023/08/CAMH_SC‐UserStory_081123pdf.pdf accessed April 21 2025.

[11] Centers for Disease Control and Prevention . 2024. Identifying Severe Maternal Morbidity (SMM) [cited 2024 August 13]. Available from: https://www.cdc.gov/maternal‐infant‐health/php/severe‐maternal‐morbidity/icd.html?CDC_AAref_Val= https://www.cdc.gov/reproductivehealth/maternalinfanthealth/smm/severe‐morbidity‐ICD.htm accessed August 13 2024.

[12] Zou, G . 2004. “A Modified Poisson Regression Approach to Prospective Studies with Binary Data.” American Journal of Epidemiology 159(7): 702–6. 10.1093/aje/kwh090. PMID: 15033648.[CrossRef] 15033648

[13] Stuart, E. A . 2010. “Matching Methods for Causal Inference: A Review and a Look Forward.” Statistical Science 25(1): 1–21. 10.1214/09-STS313. PMC2943670.[CrossRef] 20871802 PMC2943670

[14] von Hippel, P. T . 2020. “How Many Imputations Do You Need? A Two‐Stage Calculation Using a Quadratic Rule.” Sociological Methods & Research 49(3): 699–718. 39211325 10.1177/0049124117747303 PMC11361408

[15] Rubin, D. B . 1990. “Multiple imputation for nonresponse in surveys.” Computers, Environment and Urban Systems 14(1): 75. 10.1016/0198-9715(90)90061-W.

[16] Austin, P. C. , I. R. White , D. S. Lee , and S. van Buuren . 2021. “Missing Data in Clinical Research: A Tutorial on Multiple Imputation.” Canadian Journal of Cardiology 37(9): 1322–31. 10.1016/j.cjca.2020.11.010 [published Online First: 20201201]. PMC8499698.[CrossRef] 33276049 PMC8499698

[17] Smith, E. R. , E. Oakley , G. W. Grandner , K. Ferguson , F. Farooq , Y. Afshar , M. Ahlberg , et al. 2023. “Adverse Maternal, Fetal, and Newborn Outcomes among Pregnant Women with SARS‐CoV‐2 Infection: An Individual Participant Data Meta‐Analysis.” BMJ Global Health 8(1): 10.1136/bmjgh-2022-009495. PMC9895919.[CrossRef] PMC9895919 36646475

[18] Brendolin, M. , T. Fuller , M. Wakimoto , L. Rangel , G. M. Rodrigues , R. D. Rohloff , et al. 2023. “Severe Maternal Morbidity and Mortality during the COVID‐19 Pandemic: A Cohort Study in Rio de Janeiro.” IJID Regions 6: 1–6. 10.1016/j.ijregi.2022.11.004 [published Online First: 20221110]. PMC9646996.[CrossRef] 36407853 PMC9646996

[19] Piekos, S. N. , R. T. Roper , Y. M. Hwang , T. Sorensen , N. D. Price , L. Hood , and J. J. Hadlock . 2022. “The Effect of Maternal SARS‐CoV‐2 Infection Timing on Birth Outcomes: A Retrospective Multicentre Cohort Study.” Lancet Digit Health 4(2): e95–104. 10.1016/S2589-7500(21)00250-8 [published Online First: 20220113]. PMC8758153.[CrossRef] 35034863 PMC8758153

[20] Virk, S. , K. Gangu , A. Nasrullah , A. Shah , Z. Faiz , U. Khan , D. B. Jackson , et al. 2023. “Impact of COVID‐19 on Pregnancy Outcomes across Trimesters in the United States.” Biomedicines 11(11):2886. 10.3390/biomedicines11112886 [published Online First: 20231025]. PMC10669814.[CrossRef] 38001887 PMC10669814

[21] Getahun, D. , M. R. Peltier , L. D. Lurvey , J. M. Shi , D. Braun , D. A. Sacks , A. E. Burgos , et al. 2024. “Association between SARS‐CoV‐2 Infection and Adverse Perinatal Outcomes in a Large Health Maintenance Organization.” American Journal of Perinatology 41(2): 199–207. 10.1055/s-0042-1749666 [published Online First: 20220623]. 35738286

[22] Hughes, B. L. , G. J. Sandoval , T. D. Metz , R. G. Clifton , W. A. Grobman , and G. R. Saade . 2023. “First‐ or Second‐Trimester SARS‐CoV‐2 Infection and Subsequent Pregnancy Outcomes.” American Journal of Obstetrics and Gynecology 228(2): 226e1–9. 10.1016/j.ajog.2022.08.009 [published Online First: 20220813]. PMC9374493.[CrossRef] PMC9374493 35970201

[23] Cannarella, R. , R. S. Kaiyal , M. Marino , S. La Vignera , and A. Calogero 2023. “Impact of COVID‐19 on Fetal Outcomes in Pregnant Women: A Systematic Review and Meta‐Analysis.” Journal of Personalized Medicine 13(9):1337. 10.3390/jpm13091337 [published Online First: 20230830]. PMC10533032.[CrossRef] 37763105 PMC10533032

[24] Fallach, N. , Y. Segal , J. Agassy , G. Perez , A. Peretz , G. Chodick , S. Gazit , et al. 2022. “Pregnancy Outcomes after SARS‐CoV‐2 Infection by Trimester: A Large, Population‐Based Cohort Study.” PLoS ONE 17(7): e0270893. 10.1371/journal.pone.0270893 [published Online First: 20220720]. PMC9299339.[CrossRef] 35857758 PMC9299339

[25] Ghosh, R. , J. P. Gutierrez , I. de Jesus Ascencio‐Montiel , A. Juárez‐Flores , and S. M. Bertozzi . 2024. “SARS‐CoV‐2 Infection by Trimester of Pregnancy and Adverse Perinatal Outcomes: A Mexican Retrospective Cohort Study.” BMJ Open 14(4): e075928. 10.1136/bmjopen-2023-075928 [published Online First: 20240411]. PMC11015228.[CrossRef] PMC11015228 38604636

[26] CDC Maternal Infant Health . 2024. Identifying Severe Maternal Morbidity (SMM): U.S.Centers for Disease Control and Prevention. Updated May 15, 2024. https://www.cdc.gov/maternal‐infant‐health/php/severe‐maternal‐morbidity/icd.html

[27] Matsuo, K. , J. M. Green , S. A. Herrman , R. S. Mandelbaum , and J. G. Ouzounian . 2023. “Severe Maternal Morbidity and Mortality of Pregnant Patients with COVID‐19 Infection during the Early Pandemic Period in the US.” JAMA Network Open 6(4): e237149. 10.1001/jamanetworkopen.2023.7149 [published Online First: 20230403]. PMC10082398.[CrossRef] 37027160 PMC10082398

[28] Katz, D. , B. T. Bateman , K. Kjaer , D. P. Turner , N. Z. Spence , A. S. Habib , R. B. George , et al. 2021. “The Society for Obstetric Anesthesia and Perinatology Coronavirus Disease 2019 Registry: An Analysis of Outcomes among Pregnant Women Delivering during the Initial Severe Acute Respiratory Syndrome Coronavirus‐2 Outbreak in the United States.” Anesthesia & Analgesia 133(2): 462–73. 10.1213/ANE.0000000000005592. 33830956

[29] Ko, J. Y. , C. L. DeSisto , R. M. Simeone , S. Ellington , R. R. Galang , T. Oduyebo , S. M. Gilboa , et al. 2021. “Adverse Pregnancy Outcomes, Maternal Complications, and Severe Illness among US Delivery Hospitalizations with and without a Coronavirus Disease 2019 (COVID‐19) Diagnosis.” Clinical Infectious Diseases 73(Suppl 1): S24–31. 10.1093/cid/ciab344 [published Online First: 2021/05/13]. PMC8136045.[CrossRef] 33977298 PMC8136045

[30] Papageorghiou, A. T. , P. Deruelle , R. B. Gunier , S. Rauch , P. K. García‐May , M. Mhatre , M. A. Usman , et al. 2021. “Preeclampsia and COVID‐19: Results from the INTERCOVID prospective longitudinal study.” American Journal of Obstetrics and Gynecology 225(3): 289e1–17. 10.1016/j.ajog.2021.05.014 [published Online First: 20210626]. PMC8233533.[CrossRef] PMC8233533 34187688

