# Supplementary material for: Adverse pregnancy and infant outcomes by COVID‐19 infection status before and during pregnancy
Source: Pregnancy (Hoboken). 2025 Jun 30;1(4):e70051. doi: 10.1002/pmf2.70051 (PMC12834366; doi:10.1002/pmf2.70051)
Supplement: Supplementary file 1 — Supporting Information [file PMF2-1-e70051-s001.docx]

**Supplemental Table 1.** Definitions for the Exposure, Outcomes and Covariates of Interest Based on Hospitalization and Emergency Department (ED) International Classification of Diseases, Tenth Revision, Clinical Modification (10-CM) Codes or as Reported on Birth Certificates or Death Certificates

| **Exposure, Outcomes and Covariates** | **Source** | **Definition** | **ICD-10-CM codes** |
| --- | --- | --- | --- |
| **Primary Exposure** |  |  |  |
| COVID-19 infection | SC DHEC state database | Defined as the first COVID-19 diagnosis reported to SC DHEC either pre-pregnancy or during pregnancy | -- |
| **Maternal Outcomes** |  |  |  |
| HDP | BC, UB | During pregnancy | O13, O16 |
| Preeclampsia & eclampsia | UB | During pregnancy | O11.xx, O12.xx, O14.xx, O15.xx |
| Preterm delivery | BC | 22 to <37 weeks at delivery | -- |
| Placental abruption | UB | From start of pregnancy | O45.xxx |
| Postpartum hemorrhage | UB | From start of pregnancy | O72.x |
| Severe maternal morbidity (SMM) with and without transfusion | UB | 20 or 21 indicators dependent upon the inclusion of blood transfusion. From start of pregnancy up to 42 days post delivery | CDC definition^*^ |
| GDM | BC, UB | Indicated on the birth certificate or diagnosed during the second or third trimester | O24.4, O24.9 |
| cesarean delivery | BC | -- | -- |
| Fetal death | FDC | FDC present | -- |
| **Infant Outcomes** |  |  |  |
| SGA | BC | Weighing <10th percentile at birth based on biological sex, with plausible birthweights for gestational age between 22-44 weeks gestation^†^ | -- |
| NICU | BC | NICU admission | -- |
| Infant mortality | IDC | Infant died before their 1st birthday (<365 days) | -- |
| **Covariates** |  |  |  |
| Pre-pregnancy hypertension | BC, UB | Diagnosed prior to pregnancy | O10, O11, I10 |
| Pre-pregnancy diabetes | BC, UB | Diagnosed prior to pregnancy or during the first trimester | E10, E11, O24.0, O24.1, O24.3 |

Abbreviations: BC, Birth certificate; CDC, Centers for Disease Control and Prevention; C-section, Cesarian delivery; FDC, fetal death certificate; GDM, gestational diabetes mellites; HDP, hypertensive disorders of pregnancy; IDC, infant death certificate; NICU, neonatal intensive care unit; SC DHEC, South Carolina Department of Health & Environmental Control; SGA, small for gestational age; UB, inpatient hospitalization and emergency department (ED) visit encounter data.
^*^ Centers for Disease Control and Prevention. (2024, May 14). Identifying Severe Maternal Morbidity (SMM). Retrieved August 13, 2023, from <https://www.cdc.gov/maternal-infant-health/php/severe-maternal-morbidity/icd.html?CDC_AAref_Val=https://www.cdc.gov/reproductivehealth/maternalinfanthealth/smm/severe-morbidity-ICD.htm>

^†^ Alexander GR, Himes JH, Kaufman RB, Mor J, Kogan M. A United States national reference for fetal growth. Obstetrics and gynecology. 1996;87(2):163-168.

**Supplemental Table 2.** Sociodemographic and Clinical Characteristics of Deliveries in South Carolina by COVID-19 Infection Status for Matched Dataset 3, 2020-2022^*^

|  | **Matched Analysis 3** ^†, ‡, §^ | |  |
| --- | --- | --- | --- |
|  | No  COVID-19 | COVID-19  3^rd^ Trimester | **Overall**  **(unmatched)** ^‡^ |
| **Covariates** | n=4,945 | n=4,945 | n=125,091 |
| Mothers age at delivery; mean (SD) | 28.0 (5.8) | 28.0 (5.8) | 28.4 (5.8) |
| Medicaid | 3103 (62.8) | 3103 (62.8) | 72,292 (57.8) |
| Mother's race ethnicity |  |  |  |
| Non-Hispanic White | 2692 (54.4) | 2692 (54.4) | 70,142 (56.1) |
| Non-Hispanic Black | 1632 (33.0) | 1632 (33.0) | 36,763 (29.4) |
| Hispanic | 392 (7.9) | 392 (7.9) | 11,776 (9.4) |
| Other race-ethnicity | 229 (4.6) | 229 (4.6) | 6410 (5.1) |
| Mother's education |  |  |  |
| Less than high school | 637 (12.9) | 610 (12.4) | 15,798 (12.6) |
| High school | 1469 (29.9) | 1427 (29.1) | 32,975 (26.4) |
| Some college | 1118 (22.7) | 1168 (23.8) | 27,736 (22.2) |
| College graduate | 1697 (34.5) | 1707 (34.8) | 48,091 (38.4) |
| Rural residence | 1531 (31.0) | 1542 (31.2) | 37,755 (30.2) |
| Smoking (during or pre-pregnancy) | 477 (9.6) | 380 (7.7) | 10,660 (8.5) |
| Firstborn | 1564 (31.7) | 1528 (30.9) | 39,686 (31.7) |
| Previous preterm birth | 277 (5.6) | 264 (5.3) | 6350 (5.1) |
| Pre-pregnancy hypertension | 564 (11.4) | 581 (11.7) | 13,126 (10.5) |
| Pre-pregnancy diabetes | 131 (2.6) | 120 (2.4) | 2847 (2.3) |
| Pre-pregnancy BMI category |  |  |  |
| Underweight | 155 (3.2) | 128 (2.6) | 3789 (3.0) |
| Normal | 1751 (35.7) | 1596 (32.6) | 45,207 (36.1) |
| Overweight | 1235 (25.2) | 1252 (25.6) | 32,157 (25.7) |
| Obese | 1762 (35.9) | 1914 (39.1) | 42,584 (34.0) |
| **Maternal Outcomes** |  |  |  |
| C-section | 1533 (31.0) | 1619 (32.7) | 39,447 (31.5) |
| Fetal death | 13 (0.3) | 22 (0.4) | 437 (0.4) |
| GDM | 500 (10.1) | 520 (10.5) | 12,847 (10.3) |
| HDP | 919 (18.6) | 973 (19.7) | 22,587 (18.1) |
| Placental abruption | 192 (3.9) | 266 (5.4) | 6401 (5.1) |
| Postpartum hemorrhage | 172 (3.5) | 214 (4.3) | 5250 (4.2) |
| Preeclampsia & eclampsia | 433 (8.8) | 501 (10.1) | 11,012 (8.8) |
| Preterm delivery | 467 (9.4) | 485 (9.8) | 11,875 (9.5) |
| SMM without blood transfusion | 71 (1.4) | 185 (3.7) | 1749 (1.4) |
| SMM with blood transfusion | 110 (2.2) | 230 (4.7) | 2836 (2.3) |
| **Infant outcomes** |  |  |  |
| Infant mortality | 13 (0.3) | 13 (0.3) | 415 (0.3) |
| NICU admission | 352 (7.1) | 410 (8.3) | 9579 (7.7) |
| SGA | 549 (11.1) | 511 (10.4) | 12,772 (10.2) |

Abbreviations: BMI, body mass index; C-section, Cesarean birth; GDM, gestational diabetes mellitus; HDP, hypertensive disorders of pregnancy; NICU, neonatal intensive care unit; SD, standard deviation; SGA, small for gestational age; SMM, severe maternal morbidity.
^*^ Pregnancies with first diagnosis of COVID-19 during the 1^st^ and/or 2^nd^ trimester were excluded.

^†^ The COVID-19 exposure group and the non-exposed group are matched by maternal age at delivery, race-ethnicity group, Medicaid eligibility, and delivery quarter time.
^‡^ Number of missing values (Overall): education (491), smoking (44), firstborn (93), BMI category (1354), SGA (479), NICU admission (437).

Number of missing values (Analysis 3): education (52), smoking status (6), firstborn (8), BMI category (90), SGA (49), NICU admission (46)

^§^ P-values (Analysis 3): smoking, SMM without blood transfusion, SMM with blood transfusion, and placental abruption (<0.001); pre-pregnancy BMI category (0.001); postpartum hemorrhage (0.03); preeclampsia & eclampsia (0.02); and NICU (0.03)

**Supplemental Table 3.** Association Between First COVID-19 Diagnosis Pre-pregnancy or During the 1^st^ and/or 2^nd^ Trimester with Adverse Infant Outcomes, 2020-2022

|  | **Model 1^*^** | **Model 2^†^** | **Model 3^‡^** |
| --- | --- | --- | --- |
|  | **Unmatched Analysis - RR (95% CI)** | | |
| **SGA** |  |  |  |
| No history of COVID-19 | REF | REF | -- |
| COVID-19 Pre-pregnancy | **0.90 (0.85, 0.96)** | 0.95 (0.89, 1.01)^¶^ | -- |
| COVID-19 1^st^/2^nd^ trimester | **0.85 (0.79, 0.91)** | **0.88 (0.82, 0.95)**^¶^ | -- |
| **NICU** |  |  |  |
| No history of COVID-19 | REF | REF | REF |
| COVID-19 Pre-pregnancy | **1.14 (1.07, 1.22)** | **1.09 (1.02, 1.16)**^¶^ | 0.98 (0.92, 1.06)^¶^ |
| COVID-19 1^st^/2^nd^ trimester | **1.09 (1.01, 1.17)** | 1.07 (0.99, 1.15)^¶^ | 1.02 (0.94, 1.10)^¶^ |
| **Infant Mortality** |  |  |  |
| No history of COVID-19 | REF | REF | REF |
| COVID-19 Pre-pregnancy | 1.16 (0.86, 1.56) | 1.15 (0.86, 1.55) | 0.88 (0.68, 1.15)^¶^ |
| COVID-19 1^st^/2^nd^ trimester | 0.96 (0.68, 1.37) | 0.91 (0.63, 1.31) | 0.80 (0.57, 1.14)^¶^ |
|  | **Matched Analysis 1:^§^ Pre-pregnancy - RR (95% CI)** | | |
| **SGA** |  |  |  |
| No history of COVID-19 | REF | REF | -- |
| COVID-19 Pre-pregnancy | **0.86 (0.80, 0.93)** | **0.90 (0.83, 0.97)** ^¶^ | -- |
| COVID-19 1^st^/2^nd^ trimester | -- | -- | -- |
| **NICU** |  |  |  |
| No history of COVID-19 | REF | REF | REF |
| COVID-19 Pre-pregnancy | 1.08 (0.99, 1.17) | 1.04 (0.96, 1.13) ^¶^ | 0.95 (0.87, 1.03) ^¶^ |
| COVID-19 1^st^/2^nd^ trimester | -- | -- | -- |
| **Infant Mortality** |  |  |  |
| No history of COVID-19 | REF | REF | REF |
| COVID-19 Pre-pregnancy | 1.28 (0.86, 1.90) | 1.31 (0.88, 1.95) | 0.95 (0.65, 1.37) |
| COVID-19 1^st^/2^nd^ trimester | -- | -- | -- |
|  | **Matched Analysis 2:^§^ 1^st^/2^nd^ Trimester - RR (95% CI)** | | |
| **SGA** |  |  |  |
| No history of COVID-19 | REF | REF | -- |
| COVID-19 Pre-pregnancy | -- | -- | -- |
| COVID-19 1^st^/2^nd^ trimester | **0.86 (0.79, 0.95)** | **0.91 (0.82, 0.99)** ^¶^ | -- |
| **NICU** |  |  |  |
| No history of COVID-19 | REF | REF | REF |
| COVID-19 Pre-pregnancy | -- | -- | -- |
| COVID-19 1^st^/2^nd^ trimester | 1.07 (0.96, 1.18) | 1.05 (0.95, 1.16) ^¶^ | 1.01 (0.91, 1.12) ^¶^ |
| **Infant Mortality** |  |  |  |
| No history of COVID-19 | REF | REF | REF |
| COVID-19 Pre-pregnancy | -- | -- | -- |
| COVID-19 1^st^/2^nd^ trimester | 0.81 (0.52, 1.27) | 0.80 (0.52, 1.26) | **0.65 (0.43, 0.98)** |

Abbreviations: CI, confidence interval; C-section, Cesarean delivery; NICU, neonatal intensive care unit; RR, risk ratio; SGA, small for gestational age.

^*^ Model 1 is adjusted for maternal age, delivery quarter time, Medicaid, race-ethnicity, education, and rural residence.

^†^ Model 2 is adjusted for maternal age, delivery quarter time, Medicaid, race-ethnicity, education, rural residence, smoking during or pre-pregnancy, firstborn, previous preterm delivery, pre-pregnancy hypertension, pre-pregnancy diabetes, and pre-pregnancy BMI.

^‡^ Model 3 is adjusted for maternal age, delivery quarter time, Medicaid, race-ethnicity, education, rural residence, smoking during or pre-pregnancy, firstborn, previous preterm delivery, pre-pregnancy hypertension, pre-pregnancy diabetes, pre-pregnancy BMI, gestational age.

^§^ The COVID-19 exposure group and non-exposed group are matched by maternal age at delivery, race-ethnicity group, Medicaid eligibility, and delivery quarter time.

^¶^ A modified Poisson model was used due to the convergence issues with the log-binomial model.

**Supplemental Table 4.** Association Between First COVID-19 Diagnosis During the 3^rd^ Trimester (≥28 Weeks Gestational Age) with Adverse Infant Outcomes, 2020-2022^*^

|  | **Model 1^†^** | **Model 2**^‡^ | **Model 3**^§^ |  |
| --- | --- | --- | --- | --- |
|  | **Unmatched Analysis - RR (95% CI)** | | |  |
| **SGA** |  |  |  |  |
| No history of COVID-19 | REF | REF | ---- |  |
| COVID-19 during 3rd trimester | 0.98 (0.90, 1.06) | 1.01 (0.93, 1.09) ^¶^ | ---- |  |
| **NICU** |  |  |  |  |
| No history of COVID-19 | REF | REF | REF |  |
| COVID-19 during 3rd trimester | 1.06 (0.96, 1.17) | 1.04 (0.95, 1.15) ^¶^ | 1.06 (0.97, 1.17) ^¶^ |  |
| **Infant Mortality** |  |  |  |  |
| No history of COVID-19 | REF | REF | REF |  |
| COVID-19 during 3rd trimester | 0.75 (0.43, 1.31) | 0.77 (0.44, 1.34) | 0.79 (0.45, 1.37) |  |
|  | **Matched Analysis:^‖^ 3^rd^ Trimester - RR (95% CI)** | | |  |
| **SGA** | |  |  |  |
| No history of COVID-19 | | REF | REF | ---- |
| COVID-19 during 3rd trimester | | 0.93 (0.83, 1.04) | 0.96 (0.86, 1.07) | ---- |
| **NICU** | |  |  |  |
| No history of COVID-19 | | REF | REF | REF |
| COVID-19 during 3rd trimester | | **1.16 (1.01, 1.33)** | **1.16 (1.01, 1.33)** ^¶^ | 1.13 (0.99, 1.29) ^¶^ |
| **Infant Mortality** | |  |  |  |
| No history of COVID-19 | | REF | REF | REF |
| COVID-19 during 3rd trimester | | 1.00 (0.47, 2.16) | 1.04 (0.48, 2.24) | 1.01 (0.47, 2.17) |

Abbreviations: CI, confidence interval; GA gestational age; NICU, neonatal intensive care unit; RR, risk ratio; SGA, small for gestational age.

^*^ Pregnancies with first diagnosis of COVID-19 during the 1^st^ and/or 2^nd^ trimester were excluded.

^†^ Model 1 is adjusted for maternal age, delivery quarter time, Medicaid, race-ethnicity, education, and rural residence.

^‡^ Model 2 is adjusted for maternal age, delivery quarter time, Medicaid, race-ethnicity, education, rural residence, smoking during or pre-pregnancy, firstborn, previous preterm delivery, pre-pregnancy hypertension, pre-pregnancy diabetes, and pre-pregnancy BMI.

^§^ Model 3 is adjusted for maternal age, delivery quarter time, Medicaid, race-ethnicity, education, rural residence, smoking during or pre-pregnancy, firstborn, previous preterm delivery, pre-pregnancy hypertension, pre-pregnancy diabetes, pre-pregnancy BMI, and gestational age.

^‖^ The COVID-19 exposure group and the non-exposed group are matched by maternal age at delivery, race-ethnicity group, Medicaid eligibility, and delivery quarter time.

^¶^ A modified Poisson model was used due to the convergence issues with the log-binomial model.

**145,028 Pregnancies in South Carolina from 2020 through 2022**

**Matched Set 2**

**Exposed:** First reported diagnosis of COVID-19 during the 1^st^/2^nd^ trimester (n=7,955)

**Unexposed:** No history of COVID-19 (n=7,955) matched on maternal age at delivery, race-ethnicity group, Medicaid eligibility, and delivery quarter time.

**Unexposed:** No history of COVID-19 (n=7,955)

**Matched Set 1**

**Exposed:** First reported diagnosis of COVID-19 prior to pregnancy (n=10,942)

**Unexposed:** No history of COVID-19 (n=10,942) matched on maternal age at delivery, race-ethnicity group, Medicaid eligibility, and delivery quarter time.

**Population limited to 125,091 pregnancies that entered the 3rd trimester**

**Matched Set 3**

**Exposed:** First reported diagnosis of COVID-19 during the 3rd trimester (n=4,945)

**Unexposed:** No history of COVID-19 (n=4,945) matched on maternal age at delivery, race-ethnicity group, Medicaid eligibility, and delivery quarter time.

**Supplemental Figure 1. Consort Diagram**
